# Systematic review on genomic insights into antimicrobial resistance in ESKAPE pathogens

**DOI:** 10.4102/ajlm.v15i1.2893

**Published:** 2026-08-25

**Authors:** Husna F. Ibrahim, Nasiru Abdullahi, Ibrahim Yusuf, Bashir I. Waziri, Ahmad Abdulhadi, Khadija Muhammad, Maryam M. Ibrahim, Aisha A. Abdullahi, Muhammad A. Abbas, Hamisu M. Salihu

**Affiliations:** 1 Department of Microbiology and AMR Research Unit, Kano Independent Research Centre Trust (KIRCT), Kano, Nigeria; 2 Department of Genomics and Molecular Biology, Kano independent Research Centre Trust (KIRCT), Kano, Nigeria; 3 Department of Biochemistry, Faculty of Basic Medical Science, Bayero University Kano, Kano, Nigeria; 4 Department of Microbiology, Faculty of Life Science, Bayero University Kano, Kano, Nigeria; 5 Department of Human Physiology, Faculty of Basic Medical Sciences, Bayero University Kano, Kano, Nigeria; 6 Department of Epidemiology and Population Health, Kano Independent Research Centre Trust (KIRCT), Kano, Nigeria; 7 Kano Centre for Disease Control and Prevention, Kano, Nigeria; 8 Department of Community Medicine, Faculty of Medical Sciences, Bayero University Kano, Kano, Nigeria

**Keywords:** ESKAPE pathogens, antimicrobial resistance, antimicrobial resistance genes, genomic resistance patterns, global distribution

## Abstract

**Background:**

Antimicrobial resistance (AMR) is a major global public health threat. ESKAPE pathogens *(Enterococcus faecium, Staphylococcus aureus, Klebsiella pneumoniae, Acinetobacter baumannii, Pseudomonas aeruginosa*, and *Enterobacter* spp.) pose a major threat owing to resistance to last-line antibiotics. Genomic surveillance is crucial to understanding global and regional antimicrobial resistance genes (ARGs) in AMR transmission.

**Aim:**

This systematic review synthesised global genomic evidence to identify global and region-specific ARGs distribution among ESKAPE pathogens.

**Methods:**

Following PRISMA guidelines, studies published January 2019 to December 2024 were identified from PubMed, Google Scholar, and Web of Science. Eligible studies reported genomic characteristics and resistance patterns of one or more ESKAPE pathogens from any source.

**Results:**

Seventy-seven studies were included, with most originating from Asia, followed by Europe and Africa. Clinical isolates predominated *K. pneumoniae* was the most frequently investigated pathogen, followed by *S. aureus, P. aeruginosa*, and *A. baumannii*. The most reported resistance genes were *bla*CTX-M, *bla*NDM, and *bla*SHV. Distinct regional patterns of antimicrobial resistance gene (ARG) distribution were observed, with tetracycline and quinolone resistance genes prevailing in Africa and South America, and *bla*OXA variants dominating in Asia and Europe. Region-specific ARG patterns were identified through descriptive synthesis and comparative analysis of study-reported frequencies.

**Conclusion:**

This review provides a synthesised global map of ARG distribution in ESKAPE pathogens, highlighting surveillance gaps in underrepresented regions and non-clinical settings. Addressing these gaps will support targeted genomic surveillance and stewardship programmes.

**What this study adds:**

This study contributes to the body of knowledge by mapping global and regional antimicrobial resistance gene patterns in ESKAPE pathogens, identifying key surveillance gaps and informing targeted AMR monitoring and stewardship strategies.

## Introduction

### Global antimicrobial resistance burden

Antimicrobial resistance (AMR) is one of the top global public health and development threats.^1^ To define it, AMR arises when microorganisms including bacteria, fungi, parasites, and viruses adapt and evolve to the point where antimicrobial medications that were previously effective become ineffective in treating infections.^1^ Antimicrobial resistance is globally called the ‘Silent Pandemic’, requiring urgent attention and effective management.^2^ The World Health Organization (WHO) formulated the Global Action Plan on AMR (GAP-AMR) to combat the rising challenge of AMR. In line with this initiative, in 2015, the WHO launched the Global Antimicrobial Resistance and Use Surveillance System (GLASS) to bolster surveillance efforts and promote data sharing.^3^

In 2021, an estimated 4.71 million deaths worldwide were linked to bacterial AMR, with 1.14 million deaths directly attributed to it.^4^ However, recent research estimated that 1.91 million deaths attributable to AMR and 8·22 million deaths associated with AMR could occur globally in 2050.^4^

### ESKAPE pathogens: A critical challenge

The word ESKAPE is an acronym given to six nosocomial pathogens that are known for their potential for multidrug resistance and virulence: *Enterococcus faecium, Staphylococcus aureus, Klebsiella pneumoniae, Acinetobacter baumannii, Pseudomonas aeruginosa*, and *Enterobacter* spp.^5^ In 2024, the WHO published a list of pathogens of public health concern, and categorised them into critical, high-, and medium-priority levels, with carbapenem-resistant *A. baumannii*, third-generation cephalosporin-resistant Enterobacterales, and carbapenem-resistant Enterobacterales extended being part of the critical priority category, while vancomycin-resistant *E. faecium* (VRE), carbapenem-resistant *Pseudomonas aeruginosa*, along with methicillin-resistant *S. aureus* (MRSA) falls in the high-priority list.^6^ ESKAPE pathogens are known to develop multidrug resistance and virulence through three primary mechanisms: they inactivate drugs (often via an enzyme-mediated irreversible cleavage); they modify the target site of the antibiotic; and they reduce drug accumulation either by decreasing permeability or by increasing drug efflux.^7^ In addition to their multidrug resistance mechanisms, they can form biofilms that inhibit the immune response of the host and prevent antibiotics from targeting the pathogen effectively.

β-lactam antibiotics are still essential for treating infections caused by ESKAPE pathogens; however, resistance caused by β-lactamase production has become widespread. Key extended-spectrum β-lactamases (ESBLs), including TEM, SHV, and CTX-M, compromise the efficacy of β-lactams significantly. Although carbapenems are considered last-resort agents, the emergence of carbapenemase-producing bacteria carrying enzymes such as KPC, NDM, and OXA-48-like has reduced treatment options further.

Resistance to other major antibiotic classes is also prevalent among Gram-negative pathogens such as *Escherichia coli, K. pneumoniae*, and *A. baumannii*. Common tetracycline resistance determinants include *tetA, tetB, tetC, tetD*, and *tetG*. Fluoroquinolone resistance typically arises from mutations in the quinolone resistance-determining regions of *gyrA, gyrB, parC*, and *parE*, while aminoglycoside resistance is often mediated by aminoglycoside-modifying enzymes. In Gram-positive bacteria, notably *S. aureus*, methicillin resistance is driven by the *mecA* gene located in the SCCmec element, which encodes a low-affinity penicillin-binding protein.

### Role of genomic surveillance

ESKAPE pathogens pose a serious global health threat owing to their ability to evade treatment through diverse, gene-driven resistance mechanisms. Their resistance is mediated by chromosomes or by an array of mobile genetic elements, such as plasmids, transposons, and integrons, which can spread genes mediating resistance to β-lactams, carbapenems, tetracycline, and aminoglycoside resistance genes across strains and environments through horizontal gene transfer. Despite the growing threat, existing genomic studies often focus on individual pathogens or selected resistance genes, leaving gaps in our understanding of the broader genomic landscape driving resistance across the entire ESKAPE group. This fragmented approach limits the ability to develop comprehensive interventions, as it overlooks the complex interplay of resistance mechanisms and their potential for global dissemination. While phenotypic surveillance shows what type of resistance a bacterium has, genomic surveillance helps us to understand how resistance works and where it comes from. By looking closely at bacterial DNA, we can identify the exact genes responsible for resistance, show how strains are related to each other, and track the spread of high-risk bacteria and plasmids across hospitals or even countries. This makes genomic surveillance a powerful early-warning tool for detecting new and emerging AMR threats.

### Knowledge gaps and review objectives

A systematic review of genomic data from ESKAPE pathogens is essential to consolidate current knowledge, identify cross-cutting resistance determinants, and highlight regional variations in genetic profiles. Such insights can inform the development of more effective diagnostics, vaccines, and targeted therapies, while also guiding public health strategies like antimicrobial stewardship and infection control practices. Understanding the genomic diversity of these pathogens globally will not only help to predict future resistance trends but will also promote a more equitable, regionally informed response to AMR.

However, existing systematic reviews often examined AMR in ESKAPE pathogens, but must have focused primarily on phenotypic resistance profiles,^8^ single pathogen,^9^ or region.^10^ A synthesised, cross-pathogen analysis of global genomic data identifying shared and region-specific resistance determinants is lacking. To address this gap, this review aimed to synthesise global genomic data on ESKAPE pathogens and to explore both shared and region-specific resistance genes across the globe.

## Methods

### Literature search and search strategy

The systematic review was conducted according to the Preferred Reporting Items for Systematic Reviews and Meta-analyses (PRISMA) (Online Supplementary Document 2). Articles search was conducted from 09 December 2024 to 16 December 2024 in PubMed, Google Scholar, and Web of Science; only free-text search terms were used, no MeSH (Medical Subject Headings) terms or controlled vocabulary were used for the search. The search strategy employed a standardised Boolean structure to ensure consistent retrieval across databases.

To refine the search, parentheses were used to group related terms. The core search strings applied were: (‘genomic insight’ AND (‘antimicrobial resistance’ OR ‘microbial pathogenesis’) AND (‘*Staphylococcus aureus’* OR ‘*Pseudomonas aeruginosa*’ OR ‘*Klebsiella pneumoniae*’ OR ‘*Acinetobacter baumannii*’ OR ‘*Enterococcus faecium*’ OR ‘*Enterobacter cloacae*’).

Full search strings, applied filters, and the number of records retrieved from each database are presented in Online Supplementary Document 1 for transparency and reproducibility. Google Scholar was included for its large indexing of grey literature and non-PubMed-indexed scientific publications. Because Google Scholar yields very large sets, the first 1500 hits were screened.

The search was updated last on 16 December 2024. No additional automated alerts were used; however, backward and forward citation tracking was performed of included articles to identify any additional relevant studies.

The protocol was registered in the Prospective International Registry of Systematic Reviews (PROSPERO) of the National Institute of Health Research (Registration code: CRD420251237490).

### Inclusion and exclusion criteria

Our inclusion criteria were defined using the PICO framework to enhance clarity as follows:

Population (P): studies involving any of the ESKAPE Pathogens (*E. faecium, S. aureus, K. pneumoniae, A. baumannii, P. aeruginosa*, and *E. cloacae* complex) from human, animal, or environmental sources.

Intervention (I): Genotypic characterisation of AMR, including whole-genome sequencing or polymerase chain reaction (PCR)-based detection of resistance genes.

Comparison (C): Not applicable because the review was descriptive, not comparative.

Outcome (O): Reporting of AMR genes.

We included peer-reviewed primary research articles published in English between 2019 and 2024 that reported genotypic data on AMR for any of the ESKAPE pathogens. In addition, eligible studies reported at least one of the following β-lactam resistance genes: *bla*_OXA_, *bla*_NDM_, *bla*_CTX-M_, *bla*_SHV_, *bla*_TEM_, and *bla*_KPC_ or genes associated with resistance to tetracyclines, fluoroquinolones, aminoglycosides (*aac(6’)-Ib, ant(2”)-Ia*, and *aph(3’)-III*), or methicillin.

For the exclusion criteria, non-peer-reviewed literature, review papers, genomic databases, conference abstracts, and articles published in languages other than English were excluded. Also, studies presenting information on only one resistance gene were excluded because our review focused on broader genotypic resistance profiles for more meaningful interpretation and comparison across ESKAPE pathogens. Studies containing only phenotypic data were excluded.

### Study selection and data extraction

Articles retrieved through the search strategy were first exported into the Rayyan tool for duplicate removal and initial screening. Two authors (Husna F. Ibrahim and Ahmad Abdulhadi) independently screened all titles and abstracts according to predefined eligibility criteria. Although no formal inter-rater reliability statistics (such as Cohen’s Kappa) were calculated, disagreements were resolved by a third independent reviewer (Ibrahim Yusuf). Full-text screening was performed in the same manner.

Full texts of the screened publications were obtained from appropriate sources, and the data were extracted into an MS a Microsoft Excel spreadsheet under multiple headings, including country of origin, source, isolate type, number, AMR genes identified, and year of publication, among others. Additional details, such as study type, were also recorded. The full study selection process is presented in the 2020 flow diagram.

### Data synthesis

The extracted data were synthesised narratively. Studies were grouped and analysed by geographical region, isolate source, isolate type, and antimicrobial resistance gene (ARG) type. Quantitative summaries are presented as counts (*n*) and proportions (%) of studies reporting specific ARGs or findings

### Quality assessment of studies and risk of bias

The methodological quality of the included studies was assessed using the appropriate Joanna Briggs Institute (JBI) Critical Appraisal Checklist for cross-sectional studies.^11^ All required checklist questions were extracted and reported individually in the quality assessment sheet without classifying the studies into overall categories of high, moderate, or low risk of bias. For ease of presentation and consistency, Question 1 of each JBI checklist was coded as Q1, Question 2 as Q2, and so forth throughout the quality assessment table. Each item was answered as ‘Yes’, ‘No’, ‘Unclear’, or ‘Not Applicable’, in accordance with JBI guidance. A comprehensive JBI checklist is presented in Online Supplementary Document 4.

The appraisal was conducted independently by two reviewers (Husna F. Ibrahim and Ahmad Abdulhadi), and discrepancies were resolved through discussion and, when necessary, by a third reviewer (Ibrahim Yusuf). Quality assessment outcomes were not considered as exclusion criteria but rather informed the interpretation of results.

## Results

### Study selection

**FIGURE 1:**
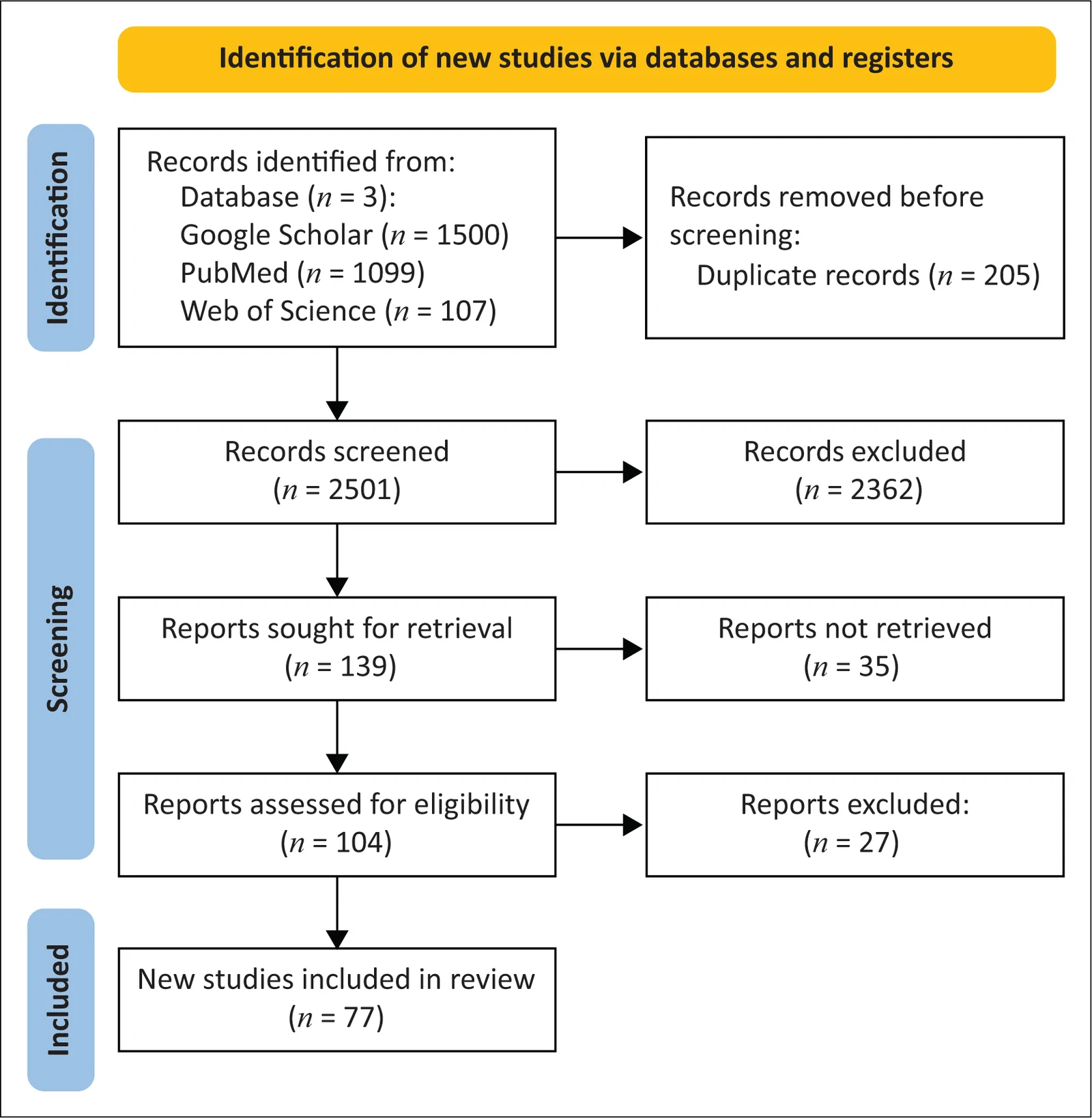
Preferred Reporting Items for Systematic Reviews and Meta-analyses flow diagram showing study selection process. The diagram shows the number of records retrieved from the searches conducted between 09 December 2024 and 16 December 2024 across all databases, duplicates removed, and the articles screened, excluded, and included in the final analysis.^12^

A thorough database search initially identified 2706 records. After removing duplicates, 2501 articles remained for initial screening. Of the 2501 articles, 2362 were excluded after reviewing their titles and abstracts. The remaining 139 articles underwent full-text screening to assess eligibility, resulting in 77 articles meeting the inclusion criteria and being included in the study (Figure 1).

### Risk of bias and quality of studies

The methodological quality of the cross-sectional studies included was assessed using the JBI Critical Appraisal Checklist for Analytical Cross-Sectional Studies. Overall, most studies satisfied the majority of the JBI quality criteria, particularly with respect to clearly defined inclusion criteria, valid and reliable measurement of exposure and outcomes, and appropriate statistical analysis. No overall risk categories (high, moderate, or low) were assigned; instead, item-level responses to each checklist question (Q1–Q8) for all studies are provided in Online Supplementary Document 3.

### Study characteristics

The study characteristics of the 77 studies included, including region of study, number of studies, isolate source, isolate type, and the ARGs detected, are shown in Table 1. A total of 77 studies were included in the review, which included 15 (19.48%) from Africa, 35 (45.45%) from Asia, 17 (22.08%) from Europe, three (3.90%) from North America, and seven (9.09%) from South America. Based on the source of isolates, about 70% (55/77) were from clinical samples, 18% (13/77) from animals, 6% (5/77) from the environment, 3% (2/77) from food, and about 1% (1/77) from each animal/environment source and environment/clinical source.

**TABLE 1:** Characteristics of studies included in this global systematic review, comprising articles published between January 2019 and December 2024, including region, number of studies, isolate source, isolate type, and antimicrobial resistance genes (ARGs) detected.

Region	No. of studies	Isolate source	Isolate type	ARGs Detected	References
Africa	4	Animal (4/15)	*K. pneumoniae*	*aac(6’)-Ib*	^13,14^
				*bla*_NDM_	
				*bla*_CTX-M_	
				*bla*_SHV_	
				*bla*_OXA-1_	
				*bla*_TEM_	
				*qnrB*	
				*tetA*	
			*P. aeruginosa*	*ant(2”)-Ia*	^15,16^
				*bla*_CTX-M_	
				*bla*_OXA-1_, *bla*_OXA-10_	
				*bla*_SHV_	
				*bla*_TEM_	
			*E. cloacae*	*bla*_CTX-M_	^14^
				*bla*_TEM_	
	10	Clinical (10/15)	*S. aureus*	*aac(6′)-Ib*	^17^
			*K. pneumoniae*	*bla*_CTX-M_	^17,18,19,20,21^
				*bla*_NDM_	
				*bla*_OXA-1_, *bla*_OXA-9_, *bla*_OXA-48_	
				*bla*_SHV_	
				*bla*_TEM_	
				*mecA*	
				*qnrB, qnrS*	
				*tetA, tetB, tetC, tetD*	
			*A. baumannii*	*bla*_NDM_	^17,22^
				*bla*_OXA-23_, *bla*_OXA-58_	
				*gyrA*	
				*parC*	
				*tetA*	
			*P. aeruginosa*	*bla*_NDM_	^17,23,24^
				*bla*_OXA-395_	
				*gyrA*	
				*qnrVC*	
				*tetG*	
			*E. cloacae*	*bla*_CTX-M_	^17^
				*bla*_OXA-48_	
	1	Environmental (1/15)	*A. baumannii*	*bla*_NDM_	^22^
				*bla*_OXA-28_, *bla*_OXA-58_	
				*gyrA*	
				*parC*	
				*tetX3*	
Asia	4	Animal (4/35)	*S. aureus*	*bla*_TEM_	^25,26,27^
				*gyrA*	
				*parC*	
				*mecA*	
				*tetM*	
			*K. pneumoniae*	*bla*_SHV_	^28^
				*qnrS*	
				*tetA*	
	28	Clinical (28/35)	*E. faecium*	*aac(6’)*	^29^
				*aph(3’)-III*	
			*S. aureus*	*aph(3’)-III*	^30,31,32,33^
				*gyrA*	
				*parC*	
				*mecA*	
				*tet38, tetC, tetK, tetM*	
			*K. pneumoniae*	*aac(6′)-Ib*	^34,35,36,37,38,39,40,41,42,43,44,45,46^
				*bla*_CTX-M_	
				*bla*_KPC_	
				*bla*_NDM_	
				*bla*_OXA-1_, *bla*_OXA-48_, *bla*_OXA-181_, *bla*_OXA-232_	
				*bla*_SHV_	
				*bla*_TEM_	
				*tetA, tetB*	
				*qnrA, qnrB, qnrS*	
			*A. baumannii*	*bla*_CTX-M_	^47,48^
				*bla*_OXA-23,_ *bla*_OXA-66,_ *bla*_OXA-69,_ *bla*_OXA-72,_ *bla*_OXA-90_	
				*tetA, tetB, tetR*	
				*qnr*	
			*P. aeruginosa*	*bla*_OXA-50_, *bla*_OXA-1202_, *bla*_OXA-1203_, *bla*_OXA-1204_	^49,50,51,52^
				*bla*_SHV_	
				*qnrB, qnrVC*	
			*E. cloacae*	*bla*_CTX-M_	^53^
				*bla*_NDM_	
				*bla*_SHV_	
				*qnrS*	
				*tetA*	
	1	Environmental (1/35)	*S. aureus*	*mecA*	^54^
	1	Environmental/Clinical (1/35)	*A. baumannii*	*bla*_OXA-23,_ *bla*_OXA-66,_ *bla*_OXA-67_	^55^
				*tet39, tetB*	
	1	Food (1/35)	*S. aureus*	*mecA*	^56^
				*tetK*	
Europe	2	Animal (2/17)	*K. pneumoniae*	*blaSHV*	^57^
			*A. baumannii*	*bla*_OXA-23_, *bla*_OXA-51_	^57^
			*P. aeruginosa*	*bla*_OXA-50_, *bla*_OXA-486_	^57,58^
			*E. cloacae*	*bla*_SHV_	^57^
				*bla*_TEM_	
		Animal or Environmental (1/17)	*E. faecium*	*tetM, tetL*	^59^
	10	Clinical (10/17)	*S. aureus*	*mecA*	^60,61^
				*tetM*	
			*K. pneumoniae*	*aac(6′)-Ib*	^61,62,63,64,65^
				*bla*_CTX-M_	
				*bla*_KPC_	
				*bla*_NDM_	
				*bla*_OXA-1_, *bla*_OXA-48_	
				*bla*_SHV_	
				*bla*_TEM_	
				*gyrA*	
				*parC*	
				*qnrB, qnrS*	
			*A. baumannii*	*aac(6′)-Ib*	^61,66,67^
				*bla*_NDM_	
				*bla*_OXA-23_, *bla*_OXA-24/40_, *bla*_OXA-51_, *bla*_OXA-58_, *bla*_OXA-66_, *bla*_OXA-72_	
				*gyrA*	
				*parC*	
				*tetB*	
			*P. aeruginosa*	*bla*_NDM_	^61,68,69^
				*bla*_OXA-395_	
				*gyrA*	
				*qnrVC*	
				*tetG*	
	3	Environmental (3/17)	*E. faecium*	*tetM*	^70^
			*K. pneumoniae*	*aac(6′)-Ib*	^71,72^
				*bla*_CTX-M_	
				*bla*_KPC_	
				*bla*_NDM_	
				*bla*_SHV_	
				*qnrB*	
				*tetA, tetB, tetD, tetR*	
	1	Food (1/17)	*E. faecium*	*aph(3’)-III*	^73^
				*tetL, tetM*	
North America	1	Animal (1/3)	*S. aureus*	*mecA*	^74^
				*tet38, tetM*	
	2	Clinical (2/3)	*K. pneumoniae*	*bla*_CTX-M_	^75^
				*bla*_KPC_	
				*bla*_SHV_	
				*gyrA*	
				*parC*	
				*qnrB*	
			*A. baumannii*	*bla*_OXA-23-like_, *bla*_OXA-66_	^76^
				*blaTEM*	
				*tetB*	
South America	3	Animal (3/7)	*S. aureus*	*mecA*	^77,78^
				*tet38*	
			*K. pneumoniae*	*bla*_CTX-M_	^79^
				*bla*_SHV_	
				*bla*_TEM_	
				*qnrE*	
				*tetA*	
	4	Clinical (4/7)	*S. aureus*	*mecA*	^80^
				*tet38*	
			*K. pneumoniae*	*bla*_CTX-M_	^81,82^
				*bla*_KPC_	
				*bla*_NDM_	
				*bla*_OXA-1_	
				*bla*_SHV_	
				*bla*_TEM_	
				*tetA, tetD*	
				*qnrS*	
			*A. baumannii*	*bla*_OXA-23_, *bla*_OXA-51-like_, *bla*_OXA-72_	^83^
				*bla*_TEM_	

Note: Please see the full reference list of Ibrahim HF, Abdullahi N, Yusuf I, et al. Systematic review on genomic insights into antimicrobial resistance in ESKAPE pathogens. Afr J Lab Med. 2026;15(1), a2893. https://doi.org/10.4102/ajlm.v15i1.2893.

ARG, antimicrobial resistance gene.

### Distribution of articles

Overall, 23/77 (29.87%) were published in 2024, 19/77 (24.68%) in 2023, 17/77 (23.38%) in 2022, 8/77 (10.39%) in 2021, 5/77 (6.49%) in 2020 and 4/77 (5.19%) were published in 2019.

Among the studies included, 33/77 (42.86%) were on *K. pneumoniae*, 14/77 (18.18%) on *S. aureus*, 10/77 (12.99%) on *P. aeruginosa*, 10/77 (12.99%) on *A. baumannii*, 5/77 (6.49%) on *E. faecium*, and only 1/77 (1.30%) on *E. cloacae*. However, some articles reported multiple pathogens; for example, one study reported *K. pneumoniae* and *E. cloacae* (2 isolates), another one reported on *K. pneumoniae, A baumannii, P. aeruginosa*, and *E. cloacae* (4 isolates), while only two studies (2.60%) included all ESKAPE pathogens.

### Distribution of ARGs

**FIGURE 2:**
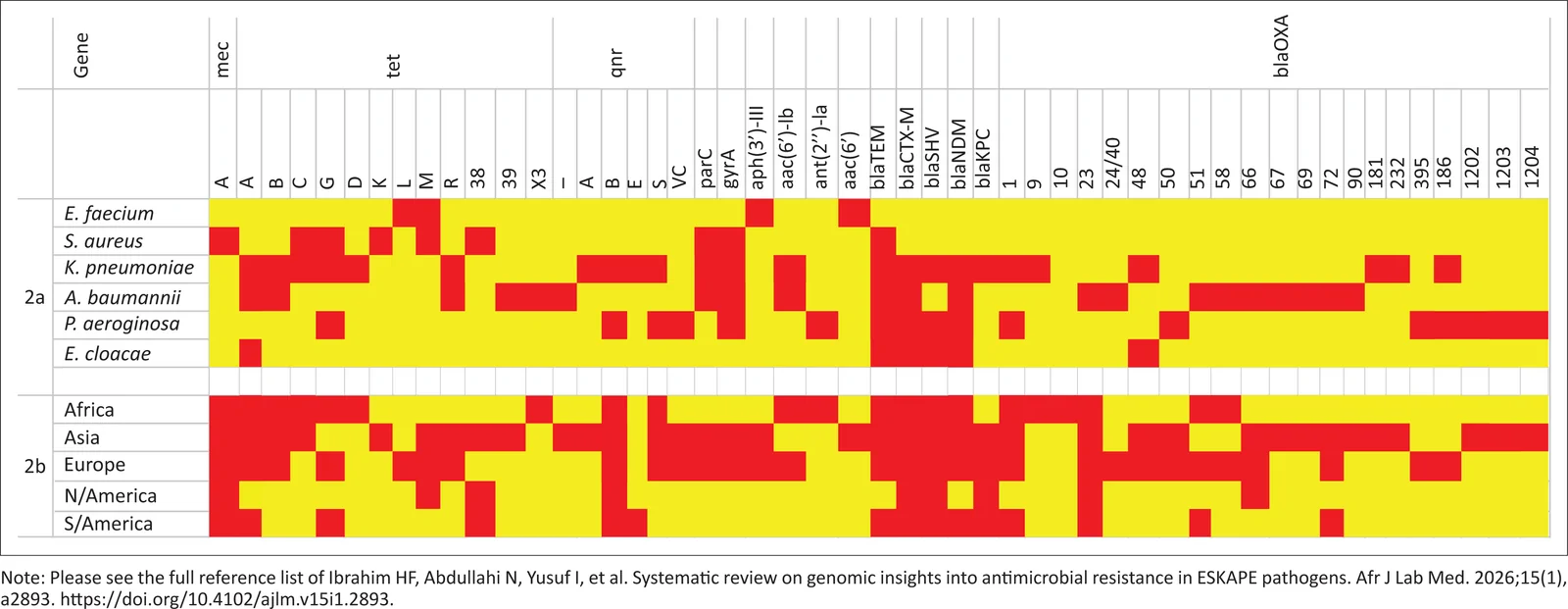
(a) Distribution of antimicrobial resistance genes among ESKAPE pathogens across global studies published between January 2019 and December 2024, showing pathogen-specific and shared genes; (b) Regional distribution of antimicrobial resistance genes among ESKAPE pathogens across global studies published between January 2019 and December 2024. Red color indicates the presence of a gene, while yellow indicates its absence.

According to the publications, *bla*_TEM_ was identified in all the ESKAPE pathogens except for *E. faecium; bla*_CTX-M_ and *bla*_NDM_ were reported in all Gram-negative ESKAPE pathogens (i.e. *K. pneumoniae, A. baumannii, P. aeruginosa*, and *E. cloacae*), while *tetM* was only reported in Gram-positive ESKAPE (*E. faecium* and *S. aureus*). For pathogen-specific ARGs, *bla*_OXA_ variants, which include 23, 24/40, 51, 58, 66, 67, 69, and 72; and *tet* variants X3 and 39 were only reported in *A. baumannii*. Similarly, *ant(2”)-Ia, bla*_OXA-50_, *bla*_OXA-395_, *bla*_OXA-1202_, *bla*_OXA-1203_ *and bla*_OXA-1204_, and *qnrVC* were specific to *P. aeruginosa*. In addition, *tet* (K and 38) and *mecA* to *S. aureus, aph(3’)-III* were only reported in *E. faecium* and *tetA* in *E. cloacae* (Figure 2a).

The β-lactams resistance encoding genes reviewed were *bla*_CTX-M_, *bla*_KPC_, *bla*_NDM_, *bla*_OXA_, *bla*_SHV_, and *bla*_TEM._ From the publications, *bla*_CTX-M_ was the most reported (*n* = 37; 48.05%), followed by *bla*_NDM_ (*n* = 35; 45.45%), *bla*_SHV_ (*n* = 32; 41.56%), *bla*_TEM_ (*n* = 22; 28.57%), and *bla*_KPC_ (*n* = 18; 23.38%). Similarly, different variants of bla_OXA_ were reported as follows: *bla*_OXA-23_ (*n* = 11; 14.29%), *bla*_OXA-1_ (*n* = 9; 11.69%), *bla*_OXA-48_ (*n* = 7; 9.09%), *bla*_OXA-1,_ *bla*_OXA-58,_ and *bla*_OXA-66_ (*n* = 4; 5.19%), *bla*_OXA-72_ (*n* = 3; 3.90%); two each of *bla*_OXA-50,_ *bla*_OXA-232,_ *bla*_OXA-395,_ and *bla*_OXA-486_ (2.60%) and the least reported were *bla*_OXA-24/40,_ *bla*_OXA-67,_ *bla*_OXA-69,_ *bla*_OXA-90,_ *bla*_OXA-181,_ *bla*_OXA-1202,_ *bla*_OXA-1203,_ and *bla*_OXA-1204_ (*n* = 1; 1.30%) (Table 2).

**TABLE 2:** Distribution of antimicrobial resistance genes based on studies included in this global systematic review (articles published between January 2019 and December 2024).

Antibiotics	ARGs	No. of studies included	Distribution (%)
β-lactams	*bla*_CTX-M_	37	48.05
	*bla*_NDM_	35	45.45
	*bla*_SHV_	32	41.56
	*bla*_TEM_	22	28.57
	*bla*_KPC_	18	23.38
	*bla*_OXA-23_	11	14.29
	*bla*_OXA-1_	9	11.69
	*bla*_OXA-48_	7	9.09
	*bla*_OXA-51_	4	5.19
	*bla*_OXA-58_	4	5.19
	*bla*_OXA-66_	4	5.19
	*bla*_OXA-72_	3	3.90
	*bla*_OXA-232_	2	2.60
	*bla*_OXA-395_	2	2.60
	*bla*_OXA-486_	2	2.60
	*bla*_OXA-50_	2	2.60
	*bla*_OXA-9_	1	1.30
	*bla*_OXA-10_	1	1.30
	*bla*_OXA-24/40_	1	1.30
	*bla*_OXA-67_	1	1.30
	*bla*_OXA-69_	1	1.30
	*bla*_OXA-90_	1	1.30
	*bla*_OXA-181_	1	1.30
	*bla*_OXA-1202_	1	1.30
	*bla*_OXA-1203_	1	1.30
	*bla*_OXA-1204_	1	1.30
Aminoglycoside	*aac(6’)-Ib*	7	9.09
	*aph(3’)-III*	2	2.60
	*ant(2”)-la*	1	1.30
	*aac(6’)*	1	1.30
Methicillin	*mecA*	16	20.78
Quinolone	*qnrB*	12	15.58
	*gyrA*	9	11.69
	*qnrS*	8	10.39
	*parC*	7	9.09
	*qnrVC*	2	2.60
	*qnrE*	1	1.30
	*qnr*	1	1.30
	*qnrA*	1	1.30
Tetracycline	*tetA*	14	18.18
	*tetB*	7	9.07
	*tetM*	5	6.49
	*tet38*	3	3.90
	*tetD*	3	3.90
	*tetK*	2	2.60
	*tetL*	2	2.60
	*tetR*	2	2.60
	*tetG*	2	2.60
	*tetC*	2	2.60
	*tet39*	1	1.30
	*tetX3*	1	1.30

Note: Please see the full reference list of Ibrahim HF, Abdullahi N, Yusuf I, et al. Systematic review on genomic insights into antimicrobial resistance in ESKAPE pathogens. Afr J Lab Med. 2026;15(1), a2893. https://doi.org/10.4102/ajlm.v15i1.2893.

ARG, antimicrobial resistance gene.

For the aminoglycoside resistance genes, *aac(6’)*-Ib was reported the most (*n* = 7; 9.09%), followed by *aph(3’)*-III (*n* = 2; 2.60%), and the least reported were *ant(2”)*-Ia, and *aac(6’)* (*n* = 1; 1.30%) (Table 2).

The *mec*A gene was reported in all 16 articles published on *S. aureus* (*n* = 16; 20.78%), other ARGs such as *tet38* and *tetM* were also reported in *S. aureus* (Table 2).

Different variants of quinolone ARGs were reported in many articles, which included *qnr, qnrA, qnrB, qnrE, qnrS, qnrVC, gyrA*, and *parC*. The most commonly reported out of these genes was *qnrB* (*n* = 12; 15.58%), followed by mutation in *gyrA* (*n* = 9; 11.69%), *qnrS* (*n* = 8; 10.39%), mutation in *parC* (*n* = 7; 9.09%), then *qnrVC* (*n* = 2; 2.60%) while the least reported genes were *qnr, qnrA*, and *qnrE* (*n* = 1; 1.30%) (Table 2).

For tetracycline, the most reported ARG was *tetA* (*n* = 14; 18.18%), followed by *tetB* (*n* = 7; 9.09%), *tetM* (*n* = 5; 6.49%), *tet38* and *tetD* (*n* = 3; 3.90%), the *tetC, tetK, tetL, tetR*, and *tetG* (*n* = 2; 2.60%), and only one study reported *tet39*, and *tetX3* in (environment, *n* = 1; 1.30%) (Table 2).

According to the studies included, the ARGs distribution across all the regions is presented in Figure 2b. However, some ARGs from the included studies were reported more often in a particular region, while some were cross-regional. *tetG* and *tetX3, ant(2”)-IIa, bla*_OXA-1,_ and *bla*_OXA-10_ were reported in studies conducted in Africa in this review. In contrast, *tetk, qnrA* and *bla*_OXA_ variants 67, 69, 90, 181, 232, 1202, 1203, and 1204 are reported in studies conducted in Asia. In addition, *tetL* and *bla*_OXA_ *variants* (24/40, 395, and 486) were found in studies conducted in Europe, while *qnrE* was only reported in South America. However, *mecA, qnrB, bla*_CTX-M_, *bla*_SHV_, and *bla*_OXA-23_ were reported in all the regions of the globe; hence, cross-regional ARGs (Figure 2b).

## Discussion

The systematic review aimed to synthesise global genomic ARG data for ESKAPE pathogens. Our analysis confirmed the widespread presence of ARGs in ESKAPE globally. It also showed that Asia has the highest number of publications on ARGs in ESKAPE pathogen. This is not surprising because Southeast Asia is regarded as a global hub for AMR emergence. After all, it has the highest risk of AMR emergence among all WHO regions in Asia, according to Shrestha, He and Legido-Quigley.^84^ The publication pattern may also be as a result of regional differences in research capacity and surveillance investment. Several Asian countries have established large AMR research networks and publish actively in journals indexed in major international databases, which may contribute to their strong representation. Following Asia, the European region has the next highest publications on ESKAPE-related ARGs, which most likely shows both the widespread distribution of the ESKAPE pathogens in the region and the emphasis of the region on AMR surveillance and research funding. Differences in indexing coverage across regions may also influence publication distribution. The African region, though predominantly low- and middle-income countries, came third as far as the number of publications on ARGs of ESKAPE pathogens is concerned. Africa has been characterised by low surveillance data on AMR owing to some challenges, which include weak laboratory infrastructure, limited capacity and training, funding and communication issues,^85^ and inability to pay for high-cost open access charges (which leads to most of the articles to be published in low impact journals).

The review indicates a notable increase in publications on AMR in ESKAPE pathogens from 2019 to 2024, with 30% of the studies published in 2024. This surge reflects heightened global attention to AMR, particularly concerning ESKAPE pathogens. The escalation in publications may be linked to the increased use of antibiotics during the coronavirus disease 2019 pandemic, which has raised concerns about the amplification of AMR.^86^ The WHO reported widespread overuse of antibiotics in hospitalised coronavirus disease 2019 patients, with approximately 75% receiving antibiotics despite only 8% having bacterial co-infections. This excessive use most likely contributed to the acceleration of AMR during and after the pandemic; hence, also in publications.^87^ In addition, some studies have indicated an increase in AMR trends during and after the coronavirus disease 2019 period compared to pre-pandemic times. For instance, a rise in resistance among pathogens such as *K. pneumoniae* and *A. baumannii* to certain antibiotics post-coronavirus disease 2019 has been reported.^88^

The systematic review revealed further that ARGs are more frequently reported in clinical samples than in non-clinical samples. The lack of adequate non-clinical data (environmental, animal, food) represents a critical knowledge gap. Antimicrobial resistance genes in clinical settings are probably part of a larger cycle involving agricultural runoff, wastewater, and wildlife. Integrating One Health surveillance using metagenomics is essential to understanding global reservoir dynamics and transmission routes. A similar trend was observed in a systematic review by Somda et al.^89^ on carbapenem-resistant Enterobacteriaceae in West Africa, which also reported a higher prevalence of resistant strains in hospital-based sources compared to environmental or community settings.

In addition, *K. pneumoniae* emerged as the most frequently reported ESKAPE pathogen globally, reinforcing its recognised role as a major driver of the global AMR burden. This aligns with previous findings by Navon-Venezia et al.,^90^ which emphasise the ability of *K. pneumoniae* to develop multi and extensive drug-resistant phenotypes, making it a critical public health threat.

Among the ARGs identified in this review, *bla*_CTX-M_, *bla*_NDM_, and *bla*_SHV_ (Table 2) were the most frequently reported. Their high prevalence is most likely because of not only their association with mobile genetic elements such as plasmids and transposons that enable rapid horizontal transfer, but also to ecological and clinical factors that promote their global dominance. For instance, *bla*_CTX-M_ variants have been linked frequently to highly successful epidemic plasmids and dominant *E. coli* and *K. pneumoniae* lineages circulating in both community and hospital environments. Also, the widespread occurrence of *bla*_NDM_ is reinforced by its repeated emergence in high-risk ESKAPE clones, which aids in its rapid spread in healthcare networks. In addition, the habit of antibiotic consumption, especially the widespread use of third-generation cephalosporins and carbapenems, leads to strong selective pressures that favour the bacteria carrying these genes. The combination of gene mobility, successful host lineages, and sustained antibiotic selection most likely explains their global dissemination and persistence.

Carbapenem resistance genes, such as *bla*_KPC_, were found exclusively in *K. pneumoniae* in this review, which aligns with findings from Teixeira et al.,^91^ who reported that *bla*_KPC_ was only detected in *K. pneumoniae*. However, this contrasts with the study by Hazen et al.,^92^ which also identified *bla*_KPC_ in *E. coli* and other bacterial species. Similarly, in this review, the bla_OXA-181_ gene was detected solely in *K. pneumoniae* strains, consistent with the findings of Somda et al.,^89^ who reported its presence in *K. pneumoniae* among Gram-negative ESKAPE pathogens studied.

The systematic review identified cross-regional ARGs as *mec*A, *qnr*B, *bla*_CTX-M_, *bla*_SHV_ and *bla*_OXA-23_ (Figure 2b), which shows their global spread of resistance genes and the potential for horizontal gene transfer. These ARGs are often found on mobile genetic elements, such as plasmids and integrons, which facilitate their rapid transfer between bacteria across different regions. This horizontal gene transfer, coupled with the widespread use of antibiotics globally, creates a strong selective pressure that promotes their persistence and dissemination worldwide.

In addition, the review identified some region-specific ARGs, such as *tetG* and *tetX3* for Africa, *tetk, qnrA*, and *bla*_OXA_ variants (67, 69, 90, 181, 232, 1202, 1203, and 1204) for Asia, *tetL*, and *bla*_OXA_ variants (24/40, 395, and 486) for Europe, and *qnrE* for South America. The reason might be that different regions often have distinct antibiotic usage patterns both in clinical and agricultural settings, which create unique selective environments. These pressures favour the acquisition and persistence of certain resistance genes over others in local bacterial populations. Moreover, regional differences in infection control practices, healthcare infrastructure, and environmental factors can influence the types of bacteria and mobile genetic elements specific to that area, thereby promoting localised genetic adaptations. This underlines the importance of regional surveillance and tailored antimicrobial stewardship strategies.

Compared with earlier systematic reviews on ESKAPE pathogens, which largely summarised phenotypic resistance^8^ or focused on specific regions^10^ or organisms,^9^ our findings highlight several novel insights. First, by incorporating only genomic studies published between 2019 and 2024, this review captures emerging ARGs that were under-represented in previous analyses. Second, integration of regional comparison demonstrates distinct ARG distributions between Africa, Asia, Europe and the Americas, a dimension not examined in earlier reviews. Finally, this synthesis integrates cross-ARGs, revealing shared ARG reservoirs and high-risk clones across ESKAPE pathogens. Together, these emphasise the added value and novel contribution of our study to the current genomic AMR landscape.

The strengths of the study include a comprehensive systematic review which incorporates a large dataset (77 studies) and provides a robust and diverse analysis of ESKAPE pathogens. It also categorises ARGs based on geographical regions, which provides insights into global and region-specific resistance trends. Finally, the review also presents detailed tables and figures that summarise ARGs distribution across different pathogens and regions, which may help in reinforcing the importance of global AMR surveillance.

These findings have direct implications for Infection Prevention and Control (IPC) and stewardship. The prevalence of *bla*_NDM_ in Asia suggests that travel screening and targeted stewardship may be warranted. The detection of identical ARG-carrying plasmids across continents emphasises the need for enhanced genomic surveillance to track transmission networks.

### Limitations

Despite its strength, the study has some limitations. It only includes studies from 2019 to 2024, which may result in excluding older but still relevant studies. The restriction to English-language studies also introduces bias, potentially overlooking non-English research from various regions. The imbalance in studies from all regions may under-represent the true AMR burden in underreported regions. In addition, the limited availability of articles from non-clinical sources may result in potential underestimation of ARG diversity in environmental and animal reservoirs.

To address these limitations, future research should focus on expanding surveillance studies to include regional or multilingual databases and non-English publications to reduce geographic and language bias. Also, incorporating metagenomics surveillance data and environmental monitoring programmes would help to capture ARGs beyond clinical settings.

### Conclusion

This systematic review provides valuable insights into the genomic basis of AMR in ESKAPE pathogens, with a strong focus on regional variations and global trends in ARGs specific to these pathogens. The study reveals important gaps in current genomic surveillance, especially in low- and middle-income countries and in non-clinical reservoirs such as the environment and clinical sectors. The findings from this review emphasise the need for continuous genomic surveillance to monitor the evolution of resistance genes, particularly in regions with a high prevalence of AMR genes.

Strengthening global data sharing, harmonising sequencing and reporting standards, and expanding surveillance capacity in underrepresented regions will be essential for improving early-warning systems for AMR threats. Also, the synthesis presented here provides an evidence base to support policymakers, researchers, and public health bodies in prioritising interventions and investing in the genomic infrastructure needed to curb the continued expansion of resistance in ESKAPE pathogens.
